# Incidence of Radiation Therapy Among Patients Enrolled in a Multidisciplinary Pulmonary Nodule and Lung Cancer Screening Clinic

**DOI:** 10.1001/jamanetworkopen.2022.4840

**Published:** 2022-03-31

**Authors:** Michael G. Milligan, Inga T. Lennes, Saif Hawari, Melin J. Khandekar, Yolonda Colson, Jo-Anne O. Shepard, Angela Frank, Lecia V. Sequist, Henning Willers, Florence K. Keane

**Affiliations:** 1 Harvard Radiation Oncology Program, Boston, Massachusetts; 2 Department of Radiation Oncology, Massachusetts General Hospital, Boston; 3 Department of Medicine, Division of Hematology-Oncology, Massachusetts General Hospital, Boston; 4 Department of Surgery, Massachusetts General Hospital, Boston; 5 Department of Radiology, Massachusetts General Hospital, Boston; 6 Department of Pulmonary and Critical Care Medicine, Massachusetts General Hospital, Boston

## Abstract

**Importance:**

The number of pulmonary nodules discovered incidentally or through screening programs has increased markedly. Multidisciplinary review and management are recommended, but the involvement of radiation oncologists in this context has not been defined.

**Objective:**

To assess the role of stereotactic body radiation therapy among patients enrolled in a lung cancer screening program.

**Design, Setting, and Participants:**

This prospective cohort study was performed at a pulmonary nodule and lung cancer screening clinic from October 1, 2012, to September 31, 2019. Referrals were based on chest computed tomography with Lung Imaging Reporting and Data System category 4 finding or an incidental nodule 6 mm or larger. A multidisciplinary team of practitioners from radiology, thoracic surgery, pulmonology, medical oncology, and radiation oncology reviewed all nodules and coordinated workup and treatment as indicated.

**Exposures:**

Patients referred to the pulmonary nodule and lung cancer screening clinic with an incidental or screen-detected pulmonary nodule.

**Main Outcomes and Measures:**

The primary outcome was the proportion of patients undergoing therapeutic intervention with radiation therapy, stratified by the route of detection of their pulmonary nodules (incidental vs screen detected). Secondary outcomes were 2-year local control and metastasis-free survival.

**Results:**

Among 1150 total patients (median [IQR] age, 66.5 [59.3-73.7] years; 665 [57.8%] female; 1024 [89.0%] non-Hispanic White; 841 [73.1%] current or former smokers), 234 (20.3%) presented with screen-detected nodules and 916 (79.7%) with incidental nodules. For patients with screen-detected nodules requiring treatment, 41 (17.5%) received treatment, with 31 (75.6%) undergoing surgery and 10 (24.4%) receiving radiation therapy. Patients treated with radiation therapy were older (median [IQR] age, 73.8 [67.1 to 82.1] vs 67.6 [61.0 to 72.9] years; *P* < .001) and more likely to have history of tobacco use (67 [95.7%] vs 128 [76.6%]; *P* = .001) than those treated with surgery. Fifty-eight patients treated with radiation therapy (82.9%) were considered high risk for biopsy, and treatment recommendations were based on a clinical diagnosis of lung cancer after multidisciplinary review. All screened patients who received radiation therapy had stage I disease and were treated with stereotactic body radiation therapy. For all patients receiving stereotactic body radiation therapy, 2-year local control was 96.3% (95% CI, 91.1%-100%) and metastasis-free survival was 94.2% (95% CI, 87.7%-100%).

**Conclusions and Relevance:**

In this unique prospective cohort, 1 in 4 patients with screen-detected pulmonary nodules requiring intervention were treated with stereotactic body radiation therapy. This finding highlights the role of radiation therapy in a lung cancer screening population and the importance of including radiation oncologists in the multidisciplinary management of pulmonary nodules.

## Key Points

**Question:**

Is there a role for radiation oncologists in the evaluation and workup of pulmonary nodules, and what is the value of radiation therapy in a lung cancer screening population?

**Findings:**

In this prospective cohort study of 1150 patients referred to a pulmonary nodule and lung cancer screening clinic, more than one-fourth of patients were recommended to undergo therapeutic intervention with surgery or radiation therapy, with most receiving treatment. Treatment was well tolerated among patients who underwent radiation therapy.

**Meaning:**

Radiation therapy is an important therapeutic modality for select patients presenting with incidental or screen-detected pulmonary nodules; therefore, radiation oncologists should be included in the multidisciplinary workup and treatment of these patients.

## Teaser

This cohort study of patients referred to a pulmonary nodule and lung cancer screening clinic assesses the value of radiation therapy and the role of radiation oncologists in this patient population.

## Introduction

The number of pulmonary nodules discovered incidentally on computed tomography (CT) has markedly increased during the past decade.^1^ In addition, with the publication of the US Preventive Services Task Force recommendations for annual lung cancer screening for high-risk individuals,^2^ the number of pulmonary nodules identified on low-dose CT (LDCT) has ballooned.^3,4^ Evaluation of pulmonary nodules requires in-depth review and care coordination, ideally in a multidisciplinary setting.^5,6^ The role of thoracic surgery is well established.^7,8,9^ However, the role of radiation oncologists in the management of pulmonary nodules is unclear, as is the value of radiation therapy (RT) in a lung cancer screening population. We describe the use of RT in a prospective cohort of 1150 patients referred to our institutional pulmonary nodule and lung cancer screening clinic (PNLCSC).

## Methods

### Study Population

**Figure 1.**
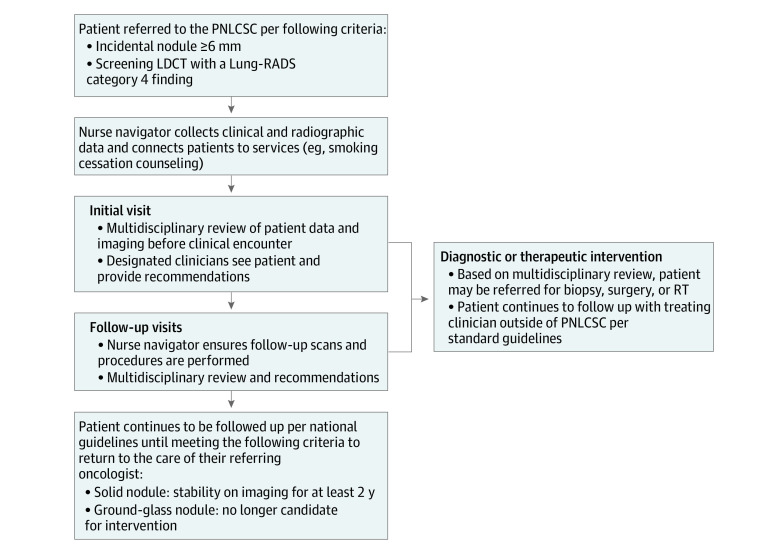
Flowchart Depicting the General Referral Criteria and Patient Flow Through the Pulmonary Nodule and Lung Cancer Screening Clinic (PNLCSC) LDCT indicates low-dose computed tomography; Lung-RADS, Lung Imaging Reporting and Data System; and RT, radiation therapy.

In this cohort study, we analyzed a prospectively collected registry of patients referred to the PNLCSC between October 1, 2012, and September 31, 2019. Radiation oncology began participating in the PNLCSC in January 2015. The structure and logistics of the clinic have been previously described^7^ and are outlined in Figure 1. Indications for referral include incidentally discovered pulmonary nodules measuring 6 mm or larger or LDCT with Lung Imaging Reporting and Data Systems category 4 finding. Clinical characteristics, interventions, and treatment outcomes were prospectively collected at the time of referral and follow-up. The data were collected prospectively for the purposes of following up patients and ensuring correct follow-up. The study was considered minimal risk, and patient consent was not required. The data were deidentified. This study was approved by the Partners Healthcare Institutional Review Board and adhered to the Strengthening the Reporting of Observational Studies in Epidemiology (STROBE) reporting guideline.

### Interventions

After multidisciplinary review, patients with pulmonary nodules suggestive of disease were recommended to undergo biopsy and/or therapeutic interventions. Patients who were not candidates for resection were referred for RT. The institutional approach to stereotactic body RT (SBRT) has been previously described.^8^ Patients for whom risk of biopsy was too high received treatment recommendations based on a clinical diagnosis of lung cancer following established criteria,^10^ requiring high-risk features, including smoking history, objective nodule growth on 3 or more successive CT scans, radiographic features consistent with malignancy, and increased fluorodeoxyglucose uptake on positron emission tomography.

After treatment, patients receiving RT were followed up at standard intervals according to National Comprehensive Cancer Network guidelines. Short-term and long-term toxic effects were graded according to Common Terminology Criteria for Adverse Events, version 4.

### Statistical Analysis

The primary outcome was the proportion of patients within the overall cohort undergoing therapeutic intervention with surgery or RT. We stratified our analysis by route of nodule detection (incidental vs screen detected) and assessed the frequency of RT among patients who required treatment before and after radiation oncology began participating in the clinic. Secondary outcomes included 2-year rates of local control and metastasis-free survival. A Fisher exact test for categorical variables and *t* test for continuous variables were used to assess clinical characteristics. Survival analyses were performed using Kaplan-Meier analysis and log-rank tests. All analyses were performed using Excel, version 2012 (Microsoft Corp) and R, version 4.1.1 (R Foundation for Statistical Computing). Significance levels were set at 2-sided *P* < .05.

## Results

### Study Population

A total of 1150 patients (median [IQR] age, 66.5 [59.3-73.7] years; 665 [57.8%] female; 3 [0.3%] American Indian or Alaska Native, 44 [3.8%] Asian, 38 [3.3%] Hispanic, 4 [0.3%] Native Hawaiian or Pacific Islander, 37 [3.2%] non-Hispanic Black or African American, and 1024 [89.0%] non-Hispanic White; 841 [73.1%] current or former smokers) were evaluated in the PNLCSC between 2012 and 2019 (Table). A total of 916 patients (79.7%) had incidental pulmonary nodules. whereas 234 (20.3%) had screen-detected nodules. Of note, the 234 patients referred for evaluation of screen-detected nodules represents 2.7% of the 8667 patients who underwent LDCT screening at our institution during the study period. Patients with screen-detected nodules were more likely to be male (369 [40.3%] vs 116 [49.6%]; *P* = .01) and have history of tobacco use (current or former smokers: 608 [66.4%] vs 234 [100%]; *P* < .001) than those with incidental nodules.

**Table.** Descriptive Baseline Characteristics of Patients Evaluated in the Pulmonary Nodule and Lung Cancer Screening Clinic Stratified

Characteristic	Incidental nodules (n = 916)	Screen-detected nodules (n = 234)	All patients (N = 1150)	*P* value
Age, median (IQR), y	66.3 (61.4-70.6)	67.4 (58.1-74.7)	66.5 (59.3-73.7)	.84
Sex, No. (%)				
Male	369 (40.3)	116 (49.6)	485 (42.2)	.01
Female	547 (59.7)	118 (50.4)	665 (57.8)	
Race, No. (%)				
American Indian or Alaska Native	3 (0.3)	0	3 (0.3)	.86
Asian	39 (4.3)	5 (2.1)	44 (3.8)	
Hispanic	38 (3.3)	9 (3.8)	38 (3.3)	
Native Hawaiian or Pacific Islander	4 (0.3)	1 (0.4)	4 (0.3)	
Non-Hispanic Black or African American	9 (3.8)	9 (3.8)	37 (3.2)	
Non-Hispanic White	814 (88.9)	209 (89.3)	1024 (89.0)	
Smoking status, No. (%)				
Current	174 (19.0)	136 (58.1)	309 (26.9)	<.001
Former	434 (47.4)	98 (41.9)	532 (46.3)	
Never	308 (33.6)	0 (0)	308 (26.8)	
Pack-years, median (IQR)	30 (16-50)	45 (32-60)	40 (20-52)	<.001
Time since quitting, median (IQR), y	22 (10-35)	9 (4-13)	19 (7-32)	<.001
Therapeutic intervention, No. (%)				
Surgery	136 (14.8)	31 (13.2)	167 (14.5)	.42
Radiotherapy	60 (6.6)	10 (4.3)	70 (6.1)	

### Therapeutic Interventions

After multidisciplinary review, 303 patients (26.4%) were recommended to undergo therapeutic intervention with surgery (218 [19.0%]) or RT (85 [7.4%]). Treatment adherence was high, with 167 of 218 (76.6%) undergoing surgery and 70 of 85 (82.4%) undergoing RT. Sixty patients underwent a biopsy before therapeutic intervention (48 of 167 [80.0%] with a biopsy before surgery and 12 of 70 [20.0%] with a biopsy before RT).

Among patients with incidental nodules requiring treatment, 136 (69.4%) underwent surgery and 60 (30.6%) RT. Among patients with screen-detected nodules requiring treatment, 31 (75.6%) underwent surgery and 10 (24.4%) RT. Before treatment, patients receiving RT were seen more times in the PNLCSC (mean number of visits: 2.68 [95% CI, 2.40-2.96] vs 1.14 [95% CI, 1.08-1.20]; *P* < .001) and had a greater number diagnostic CTs (mean number of scans: 3.19 [95% CI, 2.85-3.51] vs 1.28 [1.22-1.34]; *P* < .001).

**Figure 2.**
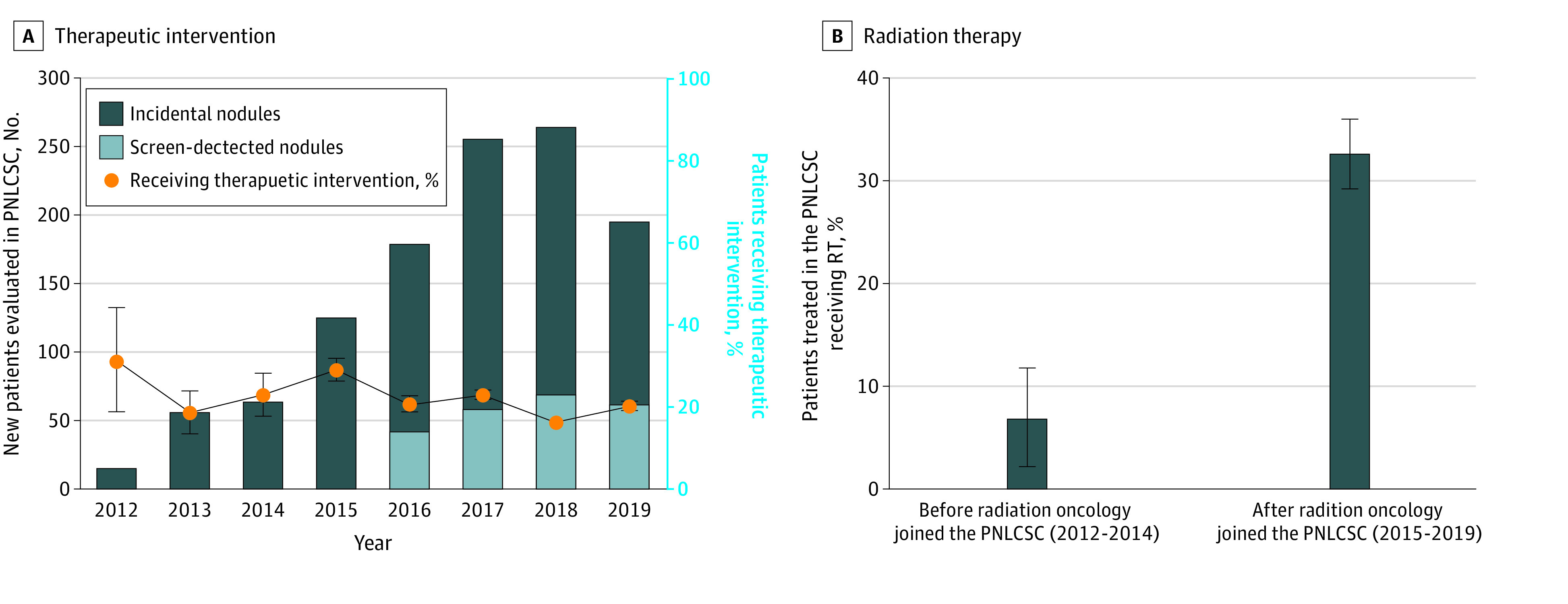
Patients Undergoing Therapeutic Intervention vs Radiation Therapy A, The number of new patients evaluated in the pulmonary nodule and lung cancer screening clinic (PNLCSC) per year and the proportion undergoing therapeutic intervention with surgery or radiation therapy. B, The proportion of treated patients receiving radiation therapy before and after radiation oncology began participating in the clinic (January 2015). Error bars indicate SEs.

Between 2012 and 2014 (before radiation oncology joined the PNLCSC), within the PNLCSC the overall rate of resection was 20.0% and the overall rate of RT was 1.5%. After the inclusion of radiation oncology, the rate of resection in the full cohort decreased to 13.8%, whereas the rate of RT increased to 6.7%. Among only the patients requiring treatment, the proportion receiving RT rather than resection increased after the inclusion of radiation oncology in the clinic (6.9% vs 32.7%; *P* = .002) (Figure 2). Compared with patients who underwent resection, patients who received RT were older (median [IQR] age, 67.6 [61.0-72.9] vs 73.8 [67.1-82.1] years; *P* < .001) and more likely to be current or former smokers (67 [95.7%] vs 128 [76.6.8%]; *P* < .001).

### Radiation Therapy

Fifty-eight patients (82.9%) treated with RT had clinically diagnosed lung cancer based on multidisciplinary review. A total of 67 patients (95.7%) were treated with SBRT to a median dose of 50 Gy (range, 48-50 Gy) in 5 fractions (range, 4-5 fractions). All screening patients who received RT were treated with SBRT.

### Outcomes

Treatment was well tolerated among patients who underwent RT. Nine patients (13.4%) experienced grade 1 to 2 toxic effects, including grade 1 to 2 dyspnea (n = 8) or chest wall pain (n = 1). No symptomatic pneumonitis or any grade 3 to 5 acute or long-term toxic effects occurred.

**Figure 3.**
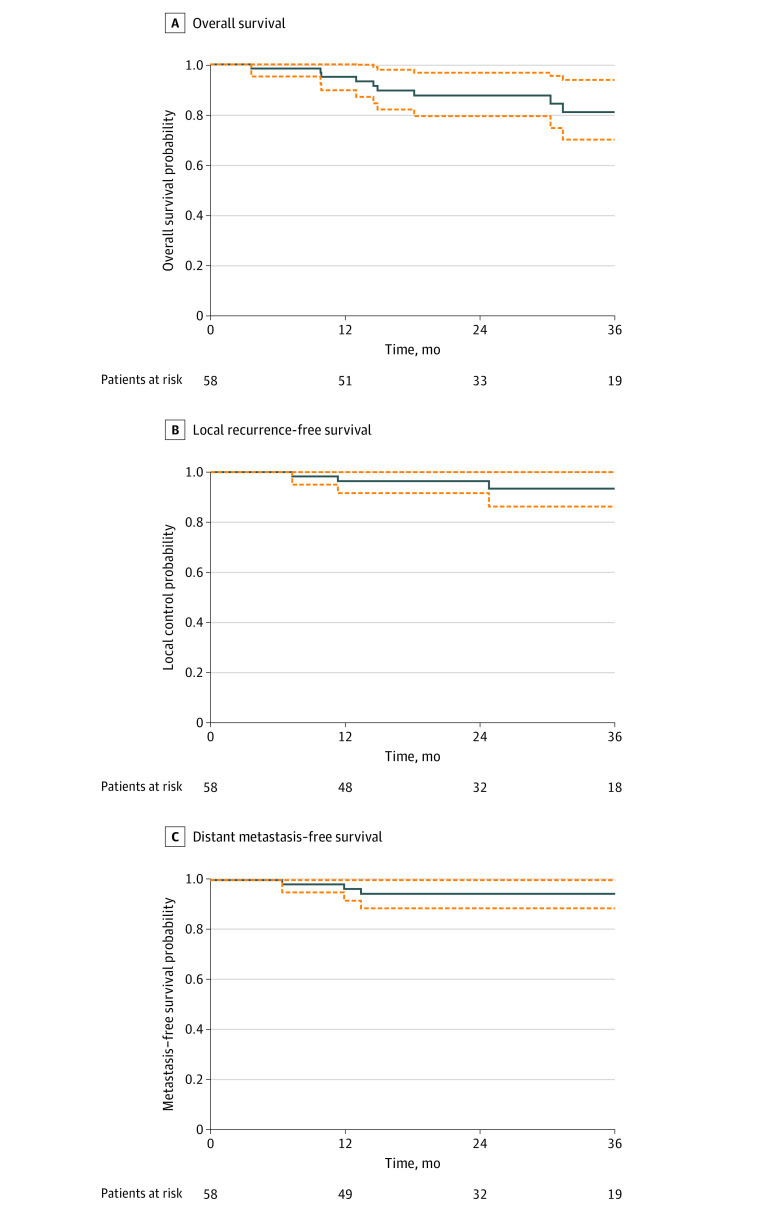
Survival of Patients Receiving Stereotactic Body Radiation Therapy (SBRT) Through the Pulmonary Nodule and Lung Cancer Screening Clinic Dashed lines indicate 95% CIs.

Median follow-up among patients undergoing SBRT was 28 months (range, 3.7-90.2 months). Two-year overall survival was 87.1% (95% CI, 78.6%-96.5%). Two-year local control was 96.2% (95% CI, 91.3%-100%). Two-year metastasis-free survival was 94.3% (95% CI, 88.3%-100%) (Figure 3).

## Discussion

As the number of patients with screen-detected or incidental pulmonary nodules continues to increase, multidisciplinary evaluation has become a widely recommend measure to ensure effective care for patients with pulmonary nodules.^5,6^ Although multidisciplinary teams have commonly included thoracic radiologists, pulmonologists, and thoracic surgeons, our experience demonstrates the critical role of radiation oncologists in workup and management of pulmonary nodules.

In our prospective cohort of 1150 patients with an incidentally detected pulmonary nodule that measured 6 mm or larger or a screening CT with a Lung Imaging Reporting and Data System category 4 finding referred to the PNLCSC, 26.4% underwent therapeutic intervention, with 19.0% undergoing surgery and 7.4% RT. The proportion of patients diagnosed with lung cancer was commensurate with experiences reported by other multidisciplinary pulmonary nodule clinics and lung cancer screening studies^11,12^ (eTable in the Supplement). As previously reported at our institution, 71% of patients with findings on LDCT suggestive of cancer are ultimately diagnosed with stage I lung cancer.^13^ However, unique to our cohort is that among screening patients who required treatment, nearly 24.4% received SBRT.

Traditionally, lung cancer screening has been recommended only for patients without comorbidities significantly limiting life expectancy.^2^ Although resection remains the standard of care for patients who are operative candidates, many patients with early-stage, screen-detected non–small cell lung cancer are medically inoperable. Among such patients, treatment is still recommended given poor outcomes associated with observation of stage I non–small cell lung cancer.^14^ In our cohort, many patients who were poor surgical candidates but deemed to have sufficient life expectancy were treated with SBRT. Our experience with SBRT for these patients was favorable, with minimal toxic effects and excellent overall survival and local control.^15^ As the general population continues to age and the number of patients who undergo screening increases, consideration of nonoperative management options is critical.

### Limitations

There are several limitations associated with our study. This study is a single-institution analysis of patients referred to a uniquely designed clinic and does not include patients seen in other settings. Our cohort includes only a small proportion of racial and ethnic minority patients, potentially reflecting disparities in access to LDCT lung cancer screening.^16^ Most patients who underwent SBRT did not have a biopsy. Although strict criteria were in place, this may have resulted in potential treatment of patients with benign lesions. A previously published surgical cohort from our institution revealed benign findings in 17.0% of resections but only 4.0% of screen-detected nodules.^9^ Overall, the rate of clinical lung cancer diagnosis in this cohort reflects a high a priori likelihood of a cancer diagnosis given stringent criteria.^5,17^

## Conclusions

These findings suggest that inclusion of radiation oncologists in a multidisciplinary pulmonary nodule clinic provides valuable expertise to optimize management of patients with both screen-detected and incidental pulmonary nodules. Stereotactic body RT represents a valuable treatment option for patients with screen-detected nodules who are not candidates for surgical resection. This finding suggests that national screening guidelines should recommend inclusion of radiation oncologists in the multidisciplinary evaluation of pulmonary nodules.

## Notes

Supplement. **eTable.** Experiences Reported by Other Multidisciplinary Pulmonary Nodule Clinics and Lung Cancer Screening Studies
