# Supplementary material for: Incidence of Radiation Therapy Among Patients Enrolled in a Multidisciplinary Pulmonary Nodule and Lung Cancer Screening Clinic
Source: JAMA Netw Open. 2022 Mar 31;5(3):e224840. doi: 10.1001/jamanetworkopen.2022.4840 (PMC8972030; doi:10.1001/jamanetworkopen.2022.4840)
Supplement: Supplement. — eTable. Experiences Reported by Other Multidisciplinary Pulmonary Nodule Clinics and Lung Cancer Screening Studies [file jamanetwopen-e224840-s001.pdf]

Supplementary Online Content

Milligan MG, Lennes IT, Hawari S, et al. Incidence of radiation therapy among patients enrolled in a multidisciplinary pulmonary nodule and lung cancer screening clinic. *JAMA Netw Open*. 2022;5(3):e224840. doi:10.1001/jamanetworkopen.2022.4840

eTable. Experiences Reported by Other Multidisciplinary Pulmonary Nodule Clinics and Lung Cancer Screening Studies

This supplementary material has been provided by the authors to give readers additional information about their work.

© 2022 Milligan MG et al. *JAMA Network Open.*

eTable. Experiences Reported by Other Multidisciplinary Pulmonary Nodule Clinics and Lung Cancer Screening Studies

Paper	Description	Inclusion Criteria	Number of Patients	Percentage of Patients with Screen-Detected Pulmonary Nodules	Radiographic Follow Up	Clinical Features Leading to Diagnostic or Therapeutic Intervention	Invasive Staging	Therapeutic Interventions	Rate of Pathologically Diagnosed Malignancy	Stages of Diagnosed Lung Cancer	Rate of Pathologically Confirmed Benign Lesions
**Prospective Lung Cancer Screening Trials**											
Crestanello, JTCS 2004	Report of the surgical experience within the Mayo CT Screening Program between 1999 to 2002	All patients were 50-years-old or older with more than a 20-pack-year smoking history. All patients underwent annual screening CT scans.	1,520	100%	Among patients who ultimately underwent surgery:<br>Thin Slice CT: 45.5%<br>Contrast-Enhanced CT: 25.5%<br>PET scan: 29.1%	Among patients who ultimately underwent surgery:<br>Growth: 58.2%<br>New nodule(s): 21.8%<br>CT enhancement: 9.1%<br>Suspicious morphology: 7.3%<br>Enlarging mediastinal nodes: 1.8%<br>Spontaneous pneumothorax: 1.8%	Among patients who ultimately underwent surgery:<br>Bronchoscopy: 9.1%<br>Percutaneous biopsy: 9.1%	Surgery: 3.6%	3.10%	I (71.1%), II (11.1%), III (15.5%), IV (2.2%)	Surgical Patients: 18.1%
Wilson, AMJRCCM 2008	Report of the outcomes of patients enrolled in the Pittsburgh Lung Screening Study between 2002 and 2005	Volunteers enrolled via advertisements and mass media into PLuSS, 50-79 years of age, no prior lung cancer, no prior chest CT within 12 months, at least 12.5 pack years of smoking history, and quit smoking no more than 10 years prior	3,624	40.6%	Among 1,477 patients with a positive finding on screening, further diagnostic thoracic CT, PET, or PET-CT was performed in 55.6%	Not Reported	Not Reported	VATS or Thoracotomy: 2.2% of full cohort, 5.6% of patients with positive screening CT finding	Full Cohort: 1.5%<br>Cohort who underwent VATS or thoracotomy: 65.9%	I (59.6%), II (3.8%), III (28.8%), IV (7.6%)	Surgical Patients: 34.1%
NLSTRT, NEJM 2011	Cohort of patients undergoing low dose CT screening as part of the National Lung Screening Trial	All LDCT patients from NLST with any positive finding on their initial or follow up scans	18,146	100%	Chest Radiograph: 14.4%<br>Chest CT: 49.8%<br>PET or PET-CT: 8.3%	Not Reported	Bronchoscopic Biopsy: 3.8%<br>Percutaneous Cytology or Biopsy: 1.8%	Surgery: 4.0%<br>Thoracoscopy: 1.3%<br>Thoracotomy: 2.9%<br>Mediastinoscopy or Mediastinotomy: 0.7%	3.6%	I (63.0%), II (7.2%), III (17.0%), IV (12.8%)	Rate of interventions not resulting in the diagnosis of lung cancer: 21%
Flores, JTCS 2014	U.S. sites of the International Early Lung Cancer Action Program trial, from 1993 to 2011.	Patients in the U.S. in I-ELCAP aged 40 to 85.	31,646	The number of positive screening exams is not reported.	Not Reported	Not Reported	Among patients who ultimately underwent surgery, 46.7% had a pre-op pathological diagnosis of lung cancer	Surgery: 1.5% of full cohort	Full Cohort: 1.4%<br>Surgical Cohort: 88.8%	I (84%), remainder not reported	Surgical Patients: 11.2%
Walker, ATS 2015	Retrospective Review of 1,654 patients screened as part of the Tufts University / Lahey Hospital Screening Program	All patients had high-risk criteria for lung cancer screening per NCCN at the time of enrollment into the study	1,654	6.3%	Among 105 patients with a positive finding on screening CT:<br>Diagnostic CT at 3 to 6 months: 49.9%<br>PET scan: 62.9%	Evidence of nodule growth on repeat CT scanning, suspicious morphology (e.g. spiculation, lobulation), PET SUV > 2.4. Rates of each finding not reported.	Among patients with positive finding:<br>Bronchoscopy: 30.5%<br>Percutaneous biopsy: 15.2%	Surgery: 1.5% of full cohort, 23.8% of patients with positive screening CT finding	Full Cohort: 1.3%<br>Surgical Cohort: 84.0%	I (61.9%), II (23.8%), III (9.5%)	Surgical Patients: 16.0%

© 2022 Milligan MG et al. *JAMA Network Open.*

Miller, Ann Thor Surg 2016	Retrospective review of a CT screening program among a hospital system in the Atlanta area between 2008 and 2013		1,267	41.0%	Not Reported	Not Reported	Among patients with positive findings:<br>Bronchoscopy: 2.3%<br>Percutaneous: 1.5%	Surgery: 1.7% of full cohort, 4.1% of patients with positive screening CT finding<br>Neoadjuvant chemo followed by surgery: 0.2% of full cohort<br>chemoradiation: 0.2% of full cohort	Full Cohort: 2.2%	I (64.3%), II (10.7%), III (17.9%), IV (7.1%)	Surgical Patients: 16.7%
**Studies on the Workup and Management of Pulmonary Nodules**											
Smith, Ann Thor Surg 2006	Retrospective review of 1,560 patients who underwent surgery for a solitary pulmonary nodule at Washington University of St. Louis from 1995 to 2002, with attention to patients pathologically confirmed to have benign disease.	All patients undergoing surgery for solitary pulmonary nodule at the institution	1,560	Prevalence of incidental or screening-detected nodules not reported	Not Reported	Not Reported	Among 140 patients found to have benign pathology, 28.5% underwent preceding needle biopsy:<br>Non-diagnostic: 20.7%<br>Negative: 3.6%<br>Positive: 2.9%	Surgery: 100%	-	Not Reported	9.0%
Veeramachaneni, JTCS 2009	Retrospective review of 414 patients with "mostly incidental pulmonary nodules" referred to dedicated nodule clinic	No specific guidelines or restrictions were placed for referral to the nodule clinic. "Highly suspicious" nodules were not typically referred, and the median nodule size was 6 mm.	414	Incidental: 72.7%<br>Screening: 27.3%	PET: 10.1% of which 19.0% of all PET scans were suggestive of malignancy	Among patients who ultimately underwent surgery:<br>Growth: 50%	Not Reported	Surgery: 4.1% of full cohort	Full Cohort: 3.1%	Not Reported	Surgical patients: 23.5%
Wiener, JAMA IM 2014	Retrospective review of 300 veterans across 15 VA centers with pulmonary nodules detected between 2003 and 2006	"Most patients had nodules that were incidental detected and did not have features associated with malignancy"	300	Not Reported	8 patients received no further workup of their pulmonary nodule due to severe comorbidities, patient preference, or in the setting of a 1-2mm nodule. 15 patients received no further workup without documented reasoning--"fell through the cracks".<br>Among the remaining 277 patients:<br>Chest X-ray: 45.1%<br>Chest CT: 77.6%<br>PET: 11.9%	Not Reported	Among all patients 15.3% underwent a biopsy procedure.	Surgery: 4.3% of full cohort	Full Cohort: 9.0%<br>Surgical Cohort: 69.2%	Not Reported	Surgical Patients: 30.7%
Melton, Cureus 2019	Retrospective review of the multidisciplinary pulmonary nodule clinic at UPMC between 2010 and 2015	All patients diagnosed with lung cancer through institutional pulmonary nodule clinic after referral for either incidental or screening-detected pulmonary nodules	119	Not Reported	Not Reported	Not Reported	Among those diagnosed with lung cancer: Percutaneous biopsy: 60.6%<br>Bronchoscopy: 11.8%<br>Surgical biopsy: 3.94%	Among all patients with a pathologic diagnosis of lung cancer, 47.9% underwent surgery	Not Reported	I (54.3%), II (13.4%), III (16.5%), IV (15.7%)	Not Reported
Verdial, Chest 2019	Retrospective review of incidentally detected pulmonary nodules at the University of Washington	Patients with incidental nodules and at 3 years of clinical follow up. Median nodule size of 8mm.	113	Incidental: 100%	Not Reported	Not Reported	Not Reported		Full Cohort: 29% lung cancer, 1.2% lung metastases	I/II (58.1%), III/IV (42.9%)	Not Reported

© 2022 Milligan MG et al. *JAMA Network Open.*

Madriaga, et al. JTCS 2020	Report of the surgical experience among patients with both incidental and screening-detected pulmonary nodules within the Pulmonary Nodule and Lung Cancer Screening Clinic at MGH between 2012 and 2018	All patients seen at the PNLCSC. Referral to the clinic was voluntary but typically limited to the following.<br>Incidental: Any suspicious nodule (typically ≥ 6mm).<br>Screening: Any LI-RADS 4 finding.	747	Incidental: 84.6%<br>Screening: 15.4%	Among patients who ultimately underwent surgery, the median number of diagnostic CTs prior to intervention were 3.0 (incidental) and 3.9 (screening), respectively. 63.5% (incidental) and 68.0% (screening) of respective patients underwent a PET scan.	Incidental: growth (91.2%), large size (3.8%), suspicious morphology (2.9%)<br>Screening: growth (76%), suspicious morphology (24%)	EBUS/Mediastinoscopy<br>Incidental: 2.9%<br>Screening: 8.0%<br>Percutaneous Biopsy<br>Incidental: 6.7%<br>Screening: 0%	Surgery: 17.3%<br>Incidental: 16.4%<br>Screening: 21.7%	Full Cohort: 14.3%<br>Incidental: 13.1%<br>Screening: 20.8%	Incidental: I (90.7%), II (2.6%), III (3.9%)<br>Screening: I (87.0%), II (4.0%), III (8.0%)	Surgical Patients: 17.1%<br>Incidental: 20.2%<br>Screening: 4.0%
LaMense, BMC Pulm Med 2020	Retrospective review of a single center's comprehensive pulmonary nodule program at a community practice in Tennessee	Patients with a new or enlarging pulmonary nodule identified either incidentally, after a symptom-directed scan, or through a screening program	1,410	Incidental: 62% in year 1, 65% in year 2	Not Reported	Not Reported	Year 1:<br>Electromagnetic navigational bronchoscopy: 21.5%<br>EBUS: 19.2%<br>Percutaneous biopsy: 4.2%<br>Mediastinoscopy: 0.4%<br>Thoracentesis: 0.8%	Year 1:<br>Surgery: 4.1% of full cohort<br>Radiation therapy: 7.3%<br>Chemotherapy: 7.1%	Year 1 cohort: 18.2%<br>Year 2 cohort: 11.5%	Year 1: I (26.4%), II (9.1%), III (26.4%), IV (38.0%)	Not Reported

© 2022 Milligan MG et al. *JAMA Network Open.*
